# Physical Performance in Older Cohorts: A Comparison of 81-Year-Old Swedish Men and Women Born Twelve Years Apart—Results from the Swedish Study “Good Aging in Skåne”

**DOI:** 10.1155/2021/8813992

**Published:** 2021-06-05

**Authors:** Henrik Ekström, Sölve Elmståhl, Lena Sandin Wranker

**Affiliations:** ^1^Division of Geriatric Medicine, Department of Clinical Sciences in Malmö, Lund University, Lund, Sweden; ^2^Centre for Ageing and Health, AGECAP, Department of Health and Rehabilitation, Institute of Neuroscience and Physiology, University of Gothenburg, Gothenburg, Sweden

## Abstract

**Materials and Methods:**

Birth cohorts of both sexes drawn from the Swedish study “Good Aging in Skåne” for the years 1920–22 and 1932–34 were compared. Walking, the step test, the chair stand test, and the handgrip strength test were used as proxies for the physical performance. The results were adjusted for lifestyle habits and common chronic geriatric diseases.

**Results:**

Both men and women in the later-born cohort walked more quickly and completed the chair stand test faster, and women were also quicker in the step test. No significant differences were found in the grip test, in either the male or female cohorts. *Discussion*. Normative reference values for physical tests of subjects of different ages can be misleading unless cohort effects are considered. Furthermore, age-related trajectories can also be misinterpreted if cohort effects are neglected which, in the longer perspective, could affect health care planning.

**Conclusion:**

Birth cohort effects should be considered when comparing walking speed, number of steps, chair stands, and the step test, in men and women of older age.

## 1. Introduction

Studies on health trends have become increasingly important in an attempt to reduce comorbidity and the number of older people in need of health care, especially in countries with a rapidly growing population of older adults [1]. One way of studying health trends at the population level is to evaluate physical performance and functional ability in different age cohorts. Studies in which cohorts are compared are often used to prognostically monitor disease development or to evaluate the results of health interventions [2]. Various physical tests, such as walking speed [3], the chair stand test [4, 5], and the handgrip strength test [6, 7], have been used as measures of health or vitality. However, previous studies comparing older age cohorts have yielded contradictory results.

Hörder et al. found no differences in slow walking (<1 m/s) in 75-year-old male or female cohorts born 19 years apart [8]. Christensen et al. found no differences in walking speed or grip strength in cohorts of 95-year-old men born 10 years apart, while in the corresponding cohorts of women the later-born cohort exhibited higher walking speed but no difference in grip strength [9]. Wranker et al. compared cohorts of 60-year-old men and women born twelve years apart and reported improvements in walking speed in the later-born cohorts [10]. A study by Strand et al., comparing cohorts of both sexes with a mean age of 70 years (born 1920–1929) and 73 years (born 1931–1949), showed that the grip strength was improved in the later-born cohorts [11].

Few studies have investigated cohort effects on physical activity in elderly cohorts of men and women (>80 years), taking several common geriatric conditions into account. The aim of this study was thus to investigate possible cohort effects on walking speed, number of steps required to walk 15 m, the step test, chair stands, and grip strength in 81-year-old men and women born twelve years apart (1920–1922 and 1932–1934), when adjusting for lifestyle habits and common chronic geriatric diseases.

## 2. Materials and Methods

### 2.1. Study Population

**Figure 1:**
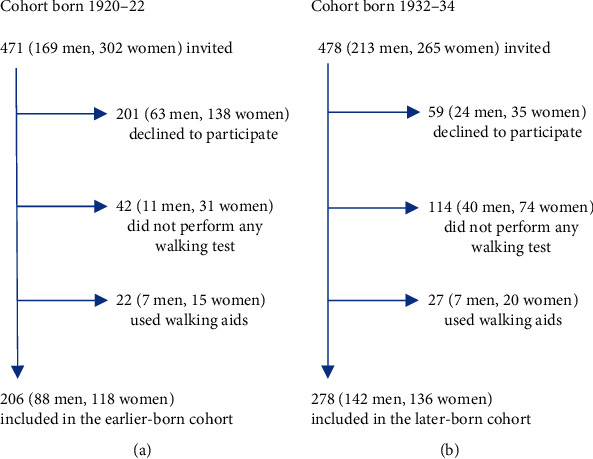
Flow sheet explaining the enrolment of men and women in the (a) first cohort born 1920–22 and in the (b) second cohort born 1932–34.

The study sample was drawn from the Good Aging in Skåne (GAS) project, a prospective, longitudinal general population study, which is part of the Swedish National Study on Ageing and Care (SNAC, http://www.snac.org) [12, 13]. The GAS project covers both urban and rural areas and includes five municipalities in the county of Skåne, the southernmost region of Sweden. Participants randomized from the National Population Register were invited to participate in the study by letter, and written informed consent was obtained from either the participants or, when necessary, from relatives. The first randomization took place between 2001 and 2004 and included a cohort born in 1920–22; the second randomization took place between 2012 and 2016 and included a cohort born in 1932–34. Out of 471 and 478 individuals invited to take part at the time of the first and second randomizations, 270 (57.3%) and 419 (87.6%) agreed to participate. The inclusion criterion was that the participant must have been able to perform at least one of the walking tests without any walking aids. This resulted in the exclusion of 205 (29.8%) individuals: 42 and 114 individuals in the first and the second cohorts had not performed any walking test, and 22 and 27 individuals in the first and second cohorts were dependent on walking aids. The final study population thus consisted of 484 participants: 206 in the first cohort born 1920–22, 88 (42.7%) men and 118 (57.3%) women), and 278 in the second cohort born 1932–34, 142 (51.3%) men and 136 (48.7%) women (Figure 1).

### 2.2. Physical Tests

The physical tests consisted of walking 2 × 15 m and measuring the time taken and the number of steps required, the step test, the chair stand test, and the handgrip strength test. These physical tests were chosen with the intention of gaining a broad idea of the participants' physical ability in terms of mobility, muscle strength, balance, and coordination. All tests were performed at the Department of Geriatrics at Malmö University Hospital. A trained registered nurse gave clear instructions on how the tests should be performed, and monitored the performance. No verbal encouragement was given during the tests. Tests were performed in one day, and the participants wore their normal clothes and shoes.

#### 2.2.1. Walking Speed and Number of Steps

Walking speed was used as a measure of functional mobility [14]. With a dynamic start, participants were asked to walk 2 × 15 m (with one 180° turn) at normal (comfortable) and maximum speed. Participants were instructed to walk 15 m, turn at a marker, return to, and pass the starting point before stopping. The test took place in a hospital corridor, and participants were allowed several meters to accelerate and decelerate before and after the test. To ensure that the participant reached the voluntary maximum in each of the maximum speed tests, the nurse gave clear instructions that the test was to be performed as quickly as possible without running. The time taken to walk 15 m and 2 × 15 m was recorded using a digital stopwatch, and the number of steps required to walk 2 × 15 m at both speeds was counted. Steps during turning were not included. Each test (normal and maximum walking speed) was performed once and participants were allowed to rest for one minute between the tests. High intraclass correlation (ICC > 0.90) has been reported for walking 15 m and 2 × 15 m at both normal and maximum speed [15]. The 2 × 15 m walking test includes turning around, which is a more complex movement which, in addition to muscle strength, also tests the individual's balance [16].

#### 2.2.2. The Step Test

This test is designed to test the subject's balance and dynamic mobility. Participants stood in front of a 7.5 cm high block placed up against a wall, with their feet parallel at a distance 5 cm from the block, and were asked to place one foot completely on the block and then return it to the floor as many times as possible in 15 seconds. The test was demonstrated by the nurse and the participant was allowed to practice once before the test started. For reasons of safety, the nurse stood close to the participant during the test, but did not support the participant [16]. Right and left lower extremities were tested separately, and the highest number of steps, for either the right or left lower extremity, was used in the analysis. This test has been found to be repeatable, valid, and reliable (ICC > 0.90) in older healthy individuals [17].

#### 2.2.3. The Chair Stand Test

The chair stand test is used to test balance, muscle strength, and sensory motor ability. Sitting on a chair with no armrests, and with the seat at a height of 45 cm, participants were asked to stand up and sit down as quickly as possible, with their arms folded across their chest and their hands on their shoulders. Before the test, and to ensure that the participants felt safe, they were asked to try to rise without using their arms [16]. Rising and sitting were first demonstrated by the nurse. The time required to stand and sit five times was recorded, and the test was performed once. High intraclass correlation has been reported for this test, ICC = 0.84 [4].

#### 2.2.4. Handgrip Strength

A device that measures the handgrip strength, Grippit®, was used for this test [18]. A standard testing procedure was used, including sitting position and instructions, as described previously [18, 19]. The handgrip device and a forearm support were mounted on a transportable base, ensuring standard arm and grip positions. The grip handle used in this study was 45 mm long, 27 mm wide, and 125 mm in circumference. The participants started to squeeze the handle on command [16]. The test was carried out twice on each hand, and the maximum force was noted. High intraclass correlation was reported (ICC = 0.97 for both hands) [20]. The best result (maximum force) was used in the analysis.

### 2.3. Sociodemographics and Lifestyle Variables

Data on sociodemography, lifestyle habits, and medical history were collected from medical and psychological examinations, self-reported questionnaires, and interviews. To control for medical history, reported diseases were verified through the National Diagnosis Registry and medical records after obtaining permission from the participants.

Sociodemographic variables included age, sex, marital status, education, and residential area. The three last variables were dichotomized: marital status into married/cohabiting or single/widowed/divorced, education into elementary school or secondary school/university, and type of residential area into rural or urban.

The lifestyle variables included were physical activity [21], divided into three categories: mostly sedentary (mostly sedentary or only light housework, including warming food, dusting, or light gardening), light activities (2–4 hours per week of housework such as cooking, vacuum cleaning, gardening, and shopping), or strenuous activities (1–3 hours per week of gymnastics, dancing, jogging, swimming, or other sports). Smoking habits were categorized as never smoked, former smoker, or current smoker, and the consumption of alcohol as never/at most once a month, 2–4 times a month, or 2–3 times a week.

### 2.4. Health Variables

Weight (in kg) was measured with a precision balance scale with light clothing and no shoes. The balance is calibrated annually by the Technical Medical Division at Skåne University Hospital. The precision of the scale was ±50 g [22]. Height was measured without shoes to the nearest 0.1 cm using a scale fixed to the wall with the participant standing erect with heels, buttocks, and shoulders against a wall and a straight fixed gaze; arms at the sides, legs straight, feet flat and heels touching each other [22]. The body mass index (BMI) was then calculated (weight (kg)/height (m^2^)).

Pain during the past month included pain in the back/pelvis, lower extremities, or upper extremities. Previously diagnosed diabetes included both type 1 and type 2 diabetes. Participants who controlled their blood sugar level by taking insulin were designated as type 1 diabetics, and those who controlled their blood sugar level by oral medication and diet were designated type 2 diabetics. Parkinson's disease, pulmonary disease (tuberculosis, asthma, chronic obstructive disease), heart disease (infarction, angina, heart failure), stroke (infarction or hemorrhage), osteoarthritis of the back, knee or hip, and fractures of vertebrae, pelvis, lower extremities, or upper extremities were classified as illness or trauma in adulthood. To confirm the presence of chronic diseases, a detailed medical examination, including records and medical history, was made by a physician.

Depressive mood was assessed using the Montgomery-Åsberg Depression Rating Scale (MADRS) including 10 questions about depressive symptoms [23]. MADRS was validated for older adults [24]. The Mini-Mental State Examination (MMSE) was used as a test of global cognitive function, on a scale from 0–30, and a score ≤ 24 was defined as an indication of cognitive impairment [25].

Anemia was defined as a hemoglobin level <120 g/l in women and <130 g/l in men [26]. The use of sedatives included regular use of any drug classified under the headings N05 or N06 in the in the Anatomical Therapeutic Chemical system, i.e., neuroleptics, tranquilizers, hypnotics, or psychoanaleptics [27]. Polypharmacy was defined as the use of 5 or more prescribed medications [28].

### 2.5. Statistical Analysis

Student's *t*-test for independent samples was applied to analyze differences between the cohorts regarding age, height, and weight. Differences in sociodemographic and lifestyle variables, pain, morbidity, use of sedatives, and MMSE score were tested with the chi-squared (*χ*^2^) test (Tables 1 and 2).

Differences in normal and maximum walking speed, number of steps, step test, chair stands, and handgrip strength between the cohorts were also tested with Student's *t*-test for independent samples. Effect size was calculated as Cohen's *d* for significant differences between the cohorts. Small effect (*d* = 0.2), moderate effect (*d* = 0.5), and large effect (*d* = 0.8) have been suggested as benchmarks [29] (Table 3).

Linear regression models were constructed with the results of the physical tests as the dependent variable and birth cohort as the independent variable, including control for confounders. Only those physical tests showing significant differences between the birth cohorts in the initial analysis were further tested in the regression models.

To reduce the risk of type II errors, only variables in the descriptive analysis with *p* values < 0.2 were included [30]. In all other analyses, *p* values < 0.05 indicated statistical significance, and all tests were two-sided. Dummy variables were constructed for BMI, smoking habits, alcohol consumption, and physical activity.

Birth cohorts and all confounding variables were entered simultaneously in the regression models. Confounders with the highest *p* value were removed one by one by examining the regression coefficients and *p* values until all the variables included showed a *p* value less than 0.1. All regression models were tested for multicollinearity, and none of the models showed a variance inflation factor >2 [31]. Normality was controlled for by inspecting histograms of the residuals, and linearity and homoscedasticity by inspecting scatter plots of standardized predicted values vs. standardized residuals. No unacceptable deviations were noted (Tables 4 and 5) [32]. All analyses were carried out using SPSS software 24 (IBM 211 Corporation, Armonk, NY, USA).

## 3. Ethics

This study, including both the earlier- and the later-born cohort, was conducted in accordance with the Declaration of Helsinki, and was approved by the Regional Ethics Committee at Lund University in 2002 (registration no. LU 744-00). All participants provided written informed consent to participate in the study, and for the retrieval of information from the National Patient Register and medical records. Participants were informed that they could withdraw from the study at any time.

## 4. Results

### 4.1. Characteristics of the Study Population

The characteristics of the men and women in each birth cohort are presented in Tables 1 and 2. Among the men, those in the later-born cohort were found to consume alcohol more often, and the prevalence of diabetes was higher. Depressive mood was less common than in the earlier-born cohort.

The women in the later-born cohort had a significantly higher weight, but no difference was found in the prevalence of diabetes. The consumption of alcohol was more frequent, and smoking had become more common. A larger proportion reported osteoarthritis, and the proportion with depressive mood had decreased.

### 4.2. Physical Tests

Among the men, all the tests except the step test and the handgrip test were performed better by the later-born cohort, although the magnitude of the effect sizes of the significant differences was moderate (*d* = 0.45–0.69). The largest differences between the cohorts were seen in the results for walking 15 m at maximum speed and in the chair stand test (Table 3). The results for walking 15 m at maximum speed and the chair stand test also showed the largest differences between the cohorts for the women. Handgrip strength was the only characteristic that did not improve in the later-born cohort, for men or women. When comparing significant differences between the cohorts, the effect sizes were moderate (*d* = 0.41–0.64) (Table 3).

The regression models revealed that the primary results remained unchanged after adjustment for significant confounders. Both men and women in the later-born cohorts were faster in the walking tests and the chair stand test. Women in the later-born cohort also performed more steps in the step test, while no significant difference was seen for the men. Handgrip strength was the only test result that did not differ between the cohorts, in men or women. In men, confounders found to be significantly associated with poorer performance were lower education and diabetes, while in women poorer performance was associated with no alcohol consumption, osteoarthritis, anemia, and an MMSE score ≤24 (Tables 4 and 5).

### 4.3. Attrition Analysis

An attrition analysis (external and internal attrition) was carried out to compare the mean age and the proportions of men and women of the participants and nonparticipants within each birth cohort. In the earlier-born cohort, the mean age of the participants was 81.0 years (SD = 0.29) and that of the nonparticipants 81.2 years (SD = 0.54) (*p* = 0.005). In the later-born cohort, the mean age of the participants was 81.0 years (SD = 0.39) and that of nonparticipants 81.2 years (SD = 0.49) (*p* = 0.001).

In the earlier-born cohort, the proportions of men and women among the participants were 42.7% (*n* = 88) and 57.3% (*n* = 118), respectively, and among the nonparticipants 28.1% (*n* = 18) and 71.9% (*n* = 46), respectively (*p* = 0.037). In the later-born cohort, the proportions of men and women among the participants were 51.1% (*n* = 142) and 48.9% (*n* = 136), respectively, and among the nonparticipants 33.3% (*n* = 47) and 66.7% (*n* = 94), respectively (*p* = 0.001) (Figure 1).

## 5. Discussion

The main result of the present study, in which 81-year-old men and women in two cohorts born twelve years apart were compared, was that the later-born cohorts performed better than the earlier-born cohorts in most of the physical tests, although the effect sizes were moderate. These results are in line with those of our previous study, in which we compared 60-year-old birth cohorts, also born twelve years apart [10]. The later-born cohorts thus outperformed the earlier-born cohorts in both studies, in the case of both men and women. More physical tests and more explanatory variables were included in the current study, especially the number of diseases, bearing in mind the high age of the participants in this study. The exception in both studies was handgrip strength, which showed no significant differences between the birth cohorts. Handgrip strength is less demanding in terms of coordination and balance [16], and the tests with greater demands on balance and coordination could have been more decisive in discriminating between the birth cohorts in the current study.

The results of the initial descriptive analyses regarding the physical tests were confirmed in the adjusted regression models. Overall, belonging to the later-born cohort was still associated with better performance. The results obtained when considering the significant confounders are somewhat ambiguous. Among men, diabetes was associated with poorer performance, although the proportion of diabetics was higher in the later-born cohort. The number of diabetics in the earlier-born cohort was small, so this finding should be interpreted with caution. The difference in the prevalence of diabetes between the two birth cohorts could perhaps be explained by the improved survival since treatment with insulin began in the early 1920s [33]. The other significant confounder, higher education, was, as might be expected, associated with better performance, and could at least partly explain some of the differences between the cohorts. Previous studies have also reported a positive relation between better health in general [34] and improved results in psychological tests [35], where education was shown to contribute to the differences between birth cohorts.

Similar reasoning can be applied to the female cohorts. In addition to belonging to the later-born cohort, modest alcohol consumption and an MMSE score >24 were associated with better performance, and a higher proportion of these variables was found in the later-born cohort. On the other hand, more frequent alcohol consumption [36] and osteoarthritis [37] were also more common in the later-born cohort of women, which could contribute to a poorer performance. Although some of the confounders seem to act in opposite directions, participants belonging to the later-born cohort still performed better, as in the case of the men.

Although smoking habits among women were not found to be a significant variable in the regression models, it is interesting to note that the proportion who never smoked was smaller in the later-born cohort. During the 1950s, when the women in the later-born cohort were of an age when many started to smoke, the proportion of women who smoked increased [38]. This could be linked to the improved social and economic status of women after World War II, when more women started to work outside the home, giving them independent incomes [39], but also as a result of the tobacco industry's campaigns in which smoking by women was portrayed as a symbol of freedom and emancipation. Women's smoking habits thus began to become more like men's [38].

However, it is difficult to identify a single health-related or sociodemographic variable that can explain the differences between the birth cohorts. The results given in the descriptive tables and by the regression models indicate that there is a clear birth cohort effect, although it cannot be ruled that other variables, individually or in combination, have been overlooked. For example, previous investigations have reported impaired walking ability in association with neurological diseases [40], as well as low skeletal muscle mass, and reduced walking speed and handgrip strength [41]. Reduced walking speed has also been shown to be related to impaired balance [42] and fear of falling [43].

It should also be borne in mind that information on lifestyle and the importance of good eating habits, reduced alcohol and tobacco consumption, and increased physical activity in relation to health has become increasingly common in recent decades, both in the media and on the Internet [44]. The proportion of older adults using the Internet is growing rapidly [45], and it cannot be ruled out that later-born cohorts have had an advantage through their more frequent use of the Internet to obtain information on health issues.

### 5.1. Strengths

The strengths of this study are that the participants were randomized from a general population including individuals living in both rural and urban areas, and that we included a relatively large number of relevant covariates.

### 5.2. Limitations

The rate of attrition is a limitation. The external and internal attrition amounted to almost 56% and 42% in the earlier-born and later-born cohorts, respectively, which could call into question the validity of the study (Figure 1). Nevertheless, such a high dropout rate is not uncommon in population studies targeting the very oldest. Although we do not know the direct causes of external attrition, deteriorating health is a likely explanation.

The internal attrition (the attrition among those who agreed to participate) accounted for almost 24% and 34% in the earlier-born and later-born cohorts, respectively. These were prospective participants that either had not completed any walking test, which was an inclusion criterion, or had used walking aids. Since it is difficult to determine the degree to which walking aids would have affected the results of the tests, we excluded these subjects. The attrition analysis comparing the mean age and the proportions of men and women within each birth cohort revealed that nonparticipants in both cohorts were slightly older (0.2 years). In both birth cohorts, and for both participants and nonparticipants, the proportion of women was higher than the proportion of men, except for participants the later-born cohort where the proportion of men was slightly higher (Figure 1). However, since the age difference between participants and nonparticipants within both cohorts was small, and the study was stratified based on gender, we believe that these differences had little or no effect on the results.

Although the external and internal attrition resulted in the most fragile individuals being excluded, and a selection bias can, therefore, not be ruled out, we believe that it is important to be aware of possible cohort effects in older men and women when assessing the results of physical tests (walking speed, number of steps, step test, chair stands, and handgrip strength). Furthermore, a higher degree of participation would probably not have affected our findings, rather the opposite, as the proportion that did not participate (the most fragile) was higher in the earlier-born cohort, 56% (*n* = 265) versus 44% (*n* = 200) (Figure 1).

Another limitation, also related to the participation rate, is the relatively small number of participants in the various diagnostic groups, which could make it difficult to assess significant differences between the cohorts. Furthermore, one could also question whether the differences we saw were the result of a birth cohort effect or a period effect. But what we intend to be a cohort effect in this study are the differences in the results of physical tests that can be seen as a period effect but which affects the responses of different birth cohorts, e.g., the results of current physical tests which can be attributed the two cohorts under study [46].

## 6. Conclusions

The cohort effects found in this study could have several implications. There is a risk that normative reference values for physical activities in different age groups, which are often derived from cross-sectional studies, are incorrect. Furthermore, age-related trajectories may be misinterpreted if confounded by cohort effects [47], which may in turn lead to uncertainties in social planning of health care, if cohort effects are not considered. It is not unlikely that the better results of the physical tests in the later-born cohort are associated with better functioning and perhaps a more active lifestyle, which could have positive effects on the general state of health, and contribute to a better quality of life. If this is the case, planning for, and facilitating, healthier lifestyles in the older population is likely to become increasingly important for community planners, not least from a salutogenic perspective.

## Figures and Tables

**Table 1:** Description of the 81-year-old men in the birth cohorts 1920–22 and 1932–34 from the Good Aging in Skåne general population study.

81-year-old men	Cohort born 1920–22, *n* = 88	Cohort born 1932–34, *n* = 142	*p* value
Year of examination	2001–2004	2013–2015	
Age (y), mean (SD)	81.1 (0.28)	81.0 (0.49)	0.264
Height (cm), mean (SD)	172.9 (6.8)	173.3 (5.5)	0.665
Weight (kg), mean (SD)	80.6 (10.9)	81.5 (11.4)	0.587
BMI			
Normal/underweight, *n* (%)	27 (30.7)	44 (31.0	
Overweight, *n* (%)	41 (46.6)	68 (47.9)	0.958
Obese, *n* (%)	20 (22.7)	30 (21.1)	
Marital status			
Married/cohabiting, *n* (%)	63 (71.6)	106 (74.6)	
Single/widowed/divorced, *n* (%)	25 (28.4)	36 (25.4)	0.610
Education			
Elementary school, *n* (%)	49 (55.7)	66 (46.5)	
Secondary school/university, *n* (%)	39 (44.3)	76 (53.5)	0.175
Type of residential area			
Rural, *n* (%)	7 (8.0)	24 (16.9)	
Urban, *n* (%)	81 (92.0)	118 (83.1)	0.053
Smoking habits			
Current smoker, *n* (%)	6 (6.8)	7 (4.9)	
Former smoker, *n* (%)	52 (59.1)	76 (53.5)	0.490
Never smoked, *n* (%)	30 (34.1)	59 (41.5)	
Alcohol consumption			
Never/at most once per month, *n* (%)	57 (64.8)	68 (47.9)	
2–4 times per month, *n* (%)	16 (18.2)	32 (22.3)	0.034
2-3 times per week, *n* (%)	15 (17.0)	42 (29.6)	
Physical activity past year			
Mostly sedentary, *n* (%)	19 (21.6)	31 (21.8)	
Light activity, *n* (%)	49 (55.7)	73 (51.4)	0.764
Strenuous activity, *n* (%)	20 (22.7)	38 (26.8)	
Pain			
Back/pelvis, *n* (%)	17 (21.5)	37 (26.1)	0.452
Lower extremities, *n* (%)	18 (22.8)	31 (21.8)	0.870
Upper extremities, *n* (%)	17 (21.5)	37 (26.5)	0.452
Comorbidity			
Pulmonary disease: tuberculosis/asthma/COPD, *n* (%)	10 (11.4)	24 (16.9)	0.250
Heart disease: infarction/angina/heart failure, *n* (%)	48 (54.5)	74 (52.1)	0.719
Stroke: infarction/hemorrhage, *n* (%)	6 (6.8)	15 (10.6)	0.338
Fractures: vertebrae/pelvis/lower extremities, *n* (%) Upper extremities, *n* (%)	3 (3.4) 12 (13.6)	12 (8.5) 21 (14.8)	0.132 0.809
Osteoarthritis: back/knee/hip, *n* (%)	25 (28.4)	47 (33.1)	0.456
Parkinson's disease, *n* (%)	1 (1.1)	3 (2.1)	1.00
Diabetes, *n* (%)	5 (5.7)	24 (16.9)	0.013
Depressive mood, *n* (%)	6 (7.1)	1 (0.8)	0.015
MMSE score < 24, *n* (%)	9 (10.5)	19 (14.1)	0.432
Anemia, *n* (%)	13 (15.5)	21 (15.0)	0.923
Medication			
Use of sedatives, *n* (%)	16 (18.2)	29 (20.4)	0.667
Polypharmacy, ≥5 prescribed medications, *n* (%)	39 (44.3)	75 (52.8)	0.210

**Table 2:** Description of the 81-year-old women in the birth cohorts 1920–22 and 1932–34 from the Good Aging in Skåne general population study.

81-year-old women	Cohort born 1920–22, *n* = 118	Cohort born 1932–34, *n* = 136	*p* value
Year of examination	2001–2004	2013–2015	
Age (y), mean (SD)	81.0 (0.36)	81.0 (0.48)	0.839
Height (cm), mean (SD)	159.5 (5.9)	160.2 (5.3)	0.333
Weight (kg), mean (SD)	66.2 (11.7)	69.2 (11.9)	0.050
BMI			
Normal/underweight, *n* (%)	53 (44.9)	42 (30.9)	
Overweight, *n* (%)	44 (37.3)	66 (48.5)	0.066
Obese, *n* (%)	21 (17.8)	28 (20.6)	
Marital status			
Married/cohabiting, *n* (%)	39 (33.3)	55 (40.4)	
Single/widowed/divorced	78 (66.7)	81 (59.6)	0.243
Education			
Elementary school, *n* (%)	75 (64.7)	76 (55.9)	
Secondary school/university, *n* (%)	41 (35.3)	60 (44.1)	0.157
Type of residential area			
Rural, *n* (%)	8 (6.8)	19 (14.0)	
Urban, *n* (%)	109 (93.2)	117 (86.0)	0.067
Smoking habits			
Current smoker, *n* (%)	12 (10.3)	8 (5.9)	
Former smoker, *n* (%)	20 (17.1)	53 (39.0)	0.001
Never smoked, *n* (%)	85 (72.6)	75 (55.1)	
Alcohol consumption			
Never/at most once per month, *n* (%)	91 (77.8)	85 (63.0)	
2–4 times per month, *n* (%)	20 (17.1)	28 (20.7)	0.009
2-3 times per week, *n* (%)	6 (5.1)	22 (16.3)	
Physical activity past year			
Mostly sedentary, *n* (%)	16 (13.8)	16 (11.9)	
Light activity, *n* (%)	70 (60.3)	83 (61.5)	0.889
Strenuous activity, *n* (%)	30 (25.9)	36 (26.7)	
Pain			
Back/pelvis, *n* (%)	40 (37.4)	38 (28.1)	0.127
Lower extremities, *n* (%)	24 (23.1)	37 (27.4)	0.446
Upper extremities, *n* (%)	20 (19.0)	32 (23.7)	0.385
Comorbidity			
Pulmonary disease: tuberculosis, asthma/COPD, *n* (%)	17 (14.5)	20 (14.8)	0.949
Heart disease: infarction/angina/heart failure, *n* (%)	43 (36.4)	37 (27.6)	0.133
Stroke: infarction/hemorrhage, *n* (%)	9 (7.6)	12 (8.9)	0.717
Fractures: vertebrae/pelvis/lower extremities, *n* (%)	13 (11.0)	17 (12.5)	0.715
Upper extremities, *n* (%)	32 (27.1)	43 (31.6)	0.433
Osteoarthritis: back/knee/hip, *n* (%)	28 (23.7)	55 (40.4)	0.005
Parkinson's disease, *n* (%)			
Diabetes, *n* (%)	8 (6.8)	9 (6.7)	0.971
Depressive mood, *n* (%)	10 (8.6)	3 (2.3)	0.029
MMSE score < 24, *n* (%)	20 (17.1)	13 (9.7)	0.084
Anemia, *n* (%)	6 (5.2)	13 (9.7)	0.167
Medication			
Use of sedatives, *n* (%)	37 (31.4)	34 (25.0)	0.260
Polypharmacy, ≥5 prescribed medications, *n* (%)	48 (40.7)	64 (47.1)	0.307

**Table 3:** Results of walking speed test, number of steps, step test, chair stand test, and handgrip strength test. Comparison between two cohorts of 81-year-olds born 1920–22 and 1932–34 stratified by sex.

Physical test	Cohort born 1920–22			Cohort born 1932–34				
*Men*	*n*	Mean	SD	*n*	Mean	SD	*p* value	Effect size^a^
Walking 15 m								
Normal speed (m/s)	88	1.20	0.19	142	1.35	0.24	<0.001	0.69
Maximum speed (m/s)	88	1.52	0.26	139	1.72	0.32	<0.001	0.68
Walking 2 × 15 m								
Normal speed (m/s)	88	1.14	0.17	142	1.25	0.23	<0.001	0.54
Maximum speed (m/s)	88	1.43	0.28	139	1.57	0.29	0.001	0.49
Number of steps 2 × 15 m								
Normal speed (*n*)	88	45.81	5.22	142	43.11	6.69	0.001	0.45
Maximum speed (*n*)	88	42.11	5.04	139	39.27	5.79	<0.001	0.52
Step test (*n*)	87	14.94	3.92	139	15.41	3.62	0.354	
Chair stands (s)	87	12.81	3.90	131	10.64	3.03	<0.01	0.62
Handgrip strength (*N*)	88	298.0	73.9	142	302.0	71.3	0.681	

*Women*								
Walking 15 m								
Normal speed (m/s)	118	1.13	0.18	136	1.25	0.26	<0.001	0.53
Maximum speed (m/s)	118	1.37	0.23	130	1.55	0.32	<0.001	0.64
Walking 2 × 15 m								
Normal speed (m/s)	118	1.08	0.16	135	1.17	0.23	0.010	0.45
Maximum speed (m/s)	117	1.28	0.21	129	1.43	0.30	<0.001	0.57
Number of steps 2 × 15 m								
Normal speed (*n*)	118	51.74	5.26	136	48.93	8.08	0.001	0.41
Maximum speed (*n*)	118	48.51	6.38	130	45.37	6.90	<0.001	0.47
Step test (*n*)	115	13.18	3.87	129	14.73	3.47	0.001	0.42
Chair stands (s)	116	14.02	5.74	122	11.38	3.21	<0.001	0.57
Handgrip strength (*N*)	118	170.5	55.7	136	180.2	53.8	0.157	

^a^Cohen's d.

**Table 4:** Adjusted linear regression models for 81-year-old men with the results of the physical tests as the dependent variables and birth cohort as the independent variable.

81-year-old men, physical tests adjusted	*n*	*B* coefficient	SE	95% CI	*p* value
Walking 15 m, normal speed (m/s)	218				
Cohort 1920–22 vs. 1932–34		0.17	0.03	0.10/0.23	<0.001
Diabetes		−0.10	0.05	−0.19/0.00	0.045
Walking 15 m, maximum speed (m/s)	215				
Cohort 1920–22 vs. 1932–34		0.22	0.04	0.14/0.31	<0.001
Education, university		0.09	0.04	0.01/0.17	0.030
Walking 2 × 15 m, normal speed (m/s)	218				
Cohort 1920–22 vs. 1932–34		0.13	0.03	0.07/0.19	<0.001
Education, university		0.07	0.03	0.01/0.13	0.028
Walking 2 × 15 m, maximum speed (m/s)	215				
Cohort 1920–22 vs. 1932–34		0.15	0.04	0.07/0.23	<0.001
Education, university		0.12	0.04	0.04/0.20	0.004
Diabetes		−0.13	0.06	−0.25/0.00	0.041
Number of steps 2 × 15 m, normal speed (*n*)	218				
Cohort 1920–22 vs. 1932–34		−3.12	0.84	−4.65/−1.47	<0.001
Education, university		−1.98	0.81	−3.57/−0.93	0.015
Diabetes		3.59	1.25	1.12/6.06	0.005
Number of steps 2 × 15 m maximum speed (*n*)	215				
Cohort 1920–22 vs. 1932–34		−3.31	0.73	−4.74/−1.87	<0.001
Education, university		−2.08	0.70	−3.47/−0.70	0.003
Diabetes		3.45	1.11	1.29/5.63	0.002
Chair stands (s)	208				
Cohort 1920–22 vs. 1932–34		−2.01	0.46	−2.92/−1.10	<0.001
Education, university		−1.06	0.45	−1.96/−0.17	0.020

All regression models were initially adjusted for education, residential area, alcohol consumption, fractures of vertebrae/pelvis/lower extremities, diabetes, and depressive mood. The birth cohort for 1920–22 was used as the reference. Only significant confounders were included in the table.

**Table 5:** Adjusted linear regression models for 81-year-old women with results of physical tests as the dependent variables and birth cohort as the independent variable.

81-year-old women, physical tests adjusted	*n*	*B* coefficient	SE	95% CI	*p* value
Walking 15 m, normal speed (m/s)	224				
Cohort 1920–22 vs. 1932–34		0.12	0.03	0.06/0.18	<0.001
Alcohol consumption 2–4 times per month		0.12	0.04	0.05/0.19	0.001
Osteoarthritis: back/knee/hip		−0.07	0.03	−0.13/0.01	0.025
Anemia		−0.15	0.05	0.95/0.25	0.007
Walking 15 m, maximum speed (m/s)	220				
Cohort 1920–22 vs. 1932–34		0.17	0.04	0.09/0.24	<0.001
Obese		−0.12	0.05	−0.22/−0.02	0.018
Stopped smoking		0.08	0.04	0.00/0.17	0.042
Alcohol consumption: 2–4 times per month		0.14	0.05	0.05/0.22	0.003
Alcohol consumption: 2-3 times per week		0.13	0.06	0.02/0.24	0.025
Osteoarthritis: back/knee/hip		−0.08	0.04	−0.28/0.01	0.037
MMSE score >24		0.17	0.06	0.06/0.28	0.004
Walking 2 × 15 m, normal speed, (m/s)	223				
Cohort 1920–22 vs. 1932–34		0.08	0.03	0.03/0.14	0.003
Obese		−0.08	0.04	−0.15/0.00	0.043
Alcohol consumption: 2–4 times per month		0.09	0.03	0.02/0.15	0.008
Osteoarthritis: back/knee/hip		−0.07	0.03	−0.13/0.02	0.012
MMSE score >24		0.10	0.04	0.01/0.18	0.024
Anemia		−0.10	0.05	−0.20/−0.01	0.038
Walking 2 × 15 m, maximum speed (m/s)	218				
Cohort 1920–22 vs. 1932–34		0.11	0.03	0.45/0.18	0.001
Obese		−0.10	0.04	−0.19/−0.15	0.022
Stopped smoking		0.08	0.04	0.00/0.15	0.043
Alcohol consumption: 2–4 times per month		0.12	0.04	0.04/0.20	0.004
Osteoarthritis: back/knee/hip		−0.12	0.03	−0.19/−0.05	0.001
MMSE score >24		0.18	0.05	0.08/0.20	<0.001
Number of steps 2 × 15 m, normal speed (*n*)	224				
Cohort 1920–22 vs. 1932–34		−3.62	0.91	−5.42/1.82	<0.001
Overweight		2.42	1.00	0.46/4.38	0.016
Obese		3.56	1.25	1.09/6.03	0.005
Alcohol consumption: 2–4 times per month		−2.24	1.10	−4.40/−0.08	0.042
Osteoarthritis: back/knee/hip		2.80	0.96	0.90/4.70	0.004
MMSE score >24		−3.32	1.42	−6.12/−0.53	0.020
Number of steps 2 × 15 m, maximum speed (*n*)	220				
Cohort 1920–22 vs. 1932–34		−2.21	0.89	−3.97/−0.45	0.014
Obese		2.59	1.11	0.41/4.77	0.020
Alcohol consumption: 2–4 times per month		−2.44	1.09	−4.58/−0.30	0.026
Alcohol consumption: 2-3 times per week		−3.73	1.34	−6.48/−0.97	0.008
Pain: back/pelvis		2.57	0.92	0.75/4.38	0.006
MMSE score >24		−3.41	1.39	−6.15/−0.66	0.015
Step test (*n*)	215				
Cohort 1920–22 vs. 1932–34		1.19	0.48	0.24/2.14	0.014
Obese		−1.70	0.60	−2.88/−0.51	0.005
Stopped smoking		1.23	0.48	0.06/1.92	0.038
Education, university		1.00	0.53	0.18/2.27	0.022
MMSE score >24		3.20	0.77	1.67/4.72	<0.001
Chair stands (s)	209				
Cohort 1920–22 vs. 1932–34		−2.35	0.56	−3.46/−1.24	<0.001
Obese		2.58	0.74	1.13/4.03	0.001
MMSE score >24		−2.95	0.87	−4.67/−1.24	0.001

All regression models were initially adjusted for BMI, education, residential area, smoking habits, alcohol consumption, pain back/pelvis, heart disease, osteoarthritis in the back/knee/hip, depressive mood, MMSE score >24, and anemia. The birth cohort for 1920–22 was used as the reference. Only significant confounders were included in the table.

## Data Availability

The authors confirm that the data supporting the findings of this study are available within the article.

## References

[1] Maresova P., Javanmardi E., Barakovic S. (2019). Consequences of chronic diseases and other limitations associated with old age-a scoping review. *BMC Public Health*.

[2] Hotta R., Makizako H., Doi T. (2018). Healthy behaviors and incidence of disability in community-dwelling elderly. *American Journal of Health Behavior*.

[3] Makizako H., Shimada H., Doi T. (2017). Age-dependent changes in physical performance and body composition in community-dwelling Japanese older adults. *Journal of Cachexia, Sarcopenia and Muscle*.

[4] Alcazar J., Losa-Reyna J., Rodriguez-Lopez C. (2018). The sit-to-stand muscle power test: an easy, inexpensive and portable procedure to assess muscle power in older people. *Experimental Gerontology*.

[5] Pinheiro P. A., Carneiro J. A. O., Coqueiro R. S., Pereira R., Fernandes M. H. (2016). “Chair stand test” as simple tool for sarcopenia screening in elderly women. *The Journal of Nutrition, Health & Aging*.

[6] Strand B. H., Cooper R., Bergland A. (2016). The association of grip strength from midlife onwards with all-cause and cause-specific mortality over 17 years of follow-up in the Tromsø Study. *Journal of Epidemiology and Community Health*.

[7] Giudici K. V., de Souto Barreto P., de Souto Barreto P., Soriano G., Rolland Y., Vellas B. (2019). Defining vitality: associations of three operational definitions of vitality with disability in instrumental activities of daily living and frailty among elderly over a 3-year follow-up (MAPT Study). *The Journal of Nutrition, Health & Aging*.

[8] Hörder H., Skoog I., Johansson L., Falk H., Frändin K. (2015). Secular trends in frailty: a comparative study of 75-year olds born in 1911-12 and 1930. *Age and Ageing*.

[9] Christensen K., Thinggaard M., Oksuzyan A. (2013). Physical and cognitive functioning of people older than 90 years: a comparison of two Danish cohorts born 10 years apart. *The Lancet*.

[10] Wranker L. S., Elmståhl S., Ekström H. (2019). Physical performance in relation to birth cohort: a comparison of 60 year old Swedish men and women born twelve years apart. *Archives of Gerontology and Geriatrics*.

[11] Strand B. H., Bergland A., Jørgensen L., Schirmer H., Emaus N., Cooper R. (2019). Do more recent born generations of older adults have stronger grip? A comparison of three cohorts of 66- to 84-year-olds in the tromsø study. *The Journals of Gerontology: Series A*.

[12] Ekström H., Elmståhl S. (2006). Pain and fractures are independently related to lower walking speed and grip strength: results from the population study “Good Ageing in Skåne”. *Acta Orthopaedica*.

[13] Lagergren M., Fratiglioni L., Hallberg I. R. (2004). A longitudinal study integrating population, care and social services data. The Swedish National study on Aging and Care (SNAC). *Aging Clinical and Experimental Research*.

[14] Kim H.-j., Park I., Lee H. J., Lee O. (2016). The reliability and validity of gait speed with different walking pace and distances against general health, physical function, and chronic disease in aged adults. *Journal of Exercise Nutrition & Biochemistry*.

[15] Jarnlo G.-B., Nordell E. (2009). Reliability of the modified figure of eight--a balance performance test for elderly women. *Physiotherapy Theory and Practice*.

[16] Bramell-Risberg E., Jarnlo G. B., Elmståhl S. (2012). Separate physical tests of lower extremities and postural control are associated with cognitive impairment. Results from the general population study Good Aging in Skåne (GÅS-SNAC). *Clinical Interventions in Aging*.

[17] Isles R. C., Choy N. L. L., Steer M., Nitz J. C. (2004). Normal values of balance tests in women aged 2080. *Journal of the American Geriatrics Society*.

[18] Hammer A., Lindmark B. (2003). Test-retest intra-rater reliability of grip force in patients with stroke. *Journal Of Rehabilitation Medicine*.

[19] Nordenskiöld U. M., Grimby G. (1993). Grip force in patients with rheumatoid arthritis and fibromyalgia and in healthy subjects. A study with the Grippit instrument. *Scandinavian Journal of Rheumatology*.

[20] Bramell-Risberg E., Jarnlo G.-B., Elmståhl S. (2010). Slowing of alternating forearm movements is associated with cognitive impairment in community-dwelling older people. *Dementia and Geriatric Cognitive Disorders*.

[21] Saltin B., Grimby G. (1968). Physiological analysis of middle-aged and old former athletes. *Circulation*.

[22] Gavriilidou N. N., Pihlsgård M., Elmståhl S. (2015). Anthropometric reference data for elderly Swedes and its disease-related pattern. *European Journal of Clinical Nutrition*.

[23] Montgomery S. A., Åsberg M. (1979). A new depression scale designed to be sensitive to change. *British Journal of Psychiatry*.

[24] Mottram P., Wilson K., Copeland J. (2000). Validation of the Hamilton depression rating scale and Montgommery and Asberg rating scales in terms of AGECAT depression cases. *International Journal of Geriatric Psychiatry*.

[25] Folstein M. F., Folstein S. E., McHugh P. R. (1975). Mini-mental state. *Journal of Psychiatric Research*.

[26] World Health Organization (WHO) (2011). *Haemoglobin Concentrations for the Diagnosis of Anaemia and Assessment of Severity*.

[27] World Health Organization (WHO) (2009). *The Anatomical Therapeutic Chemical Classification System with Defined Daily Doses-ATC/DDD*.

[28] Vetrano D. L., Villani E. R., Grande G. (2018). Association of polypharmacy with 1-year trajectories of cognitive and physical function in nursing home residents: results from a multicenter European study. *Journal of the American Medical Directors Association*.

[29] Polit D. F., Beck C. T. (2008). *Nursing Research: Generating and Assessing Evidence for Nursing Practice*.

[30] Altman D. G. (1991). *Practical Statistics for Medical Research*.

[31] Kim J. H. (2019). Multicollinearity and misleading statistical results. *Korean Journal of Anesthesiology*.

[32] Tabachnick B. G., Fidell L. S. (2007). *Using Multivariate Statistics*.

[33] Flier J. S. (2019). Starvation in the midst of plenty: reflections on the history and biology of insulin and leptin. *Endocrine Reviews*.

[34] Marmot M., Bell R. (2016). Social inequalities in health: a proper concern of epidemiology. *Annals of Epidemiology*.

[35] Lynn R. (2009). Fluid intelligence but not vocabulary has increased in Britain, 19792008. *Intelligence*.

[36] Stringhini S., Carmeli C., Jokela M. (2018). Socioeconomic status, non-communicable disease risk factors, and walking speed in older adults: multi-cohort population based study. *BMJ*.

[37] Rome K., Dixon J., Gray M., Woodley R. (2009). Evaluation of static and dynamic postural stability in established rheumatoid arthritis: exploratory study. *Clinical Biomechanics*.

[38] Amos A., Haglund M. (2000). From social taboo to “torch of freedom”: the marketing of cigarettes to women. *Tobacco Control*.

[39] Ruggles S. (2015). Patriarchy, power, and pay: the transformation of American families, 1800-2015. *Demography*.

[40] Pirker W., Katzenschlager R. (2017). Gait disorders in adults and the elderly: a clinical guide. *Wiener Klinische Wochenschrift*.

[41] Cruz-Jentoft A. J., Baeyens J. P., Bauer J. M. (2010). Sarcopenia: European consensus on definition and diagnosis: report of the European working group on sarcopenia in older people. *Age and Ageing*.

[42] Xie Y. J., Liu E. Y., Anson E. R., Agrawal Y. (2017). Age-related imbalance is associated with slower walking speed: an analysis from the national health and nutrition examination survey. *Journal of Geriatric Physical Therapy*.

[43] Makino K., Makizako H., Doi T. (2017). Fear of falling and gait parameters in older adults with and without fall history. *Geriatrics & Gerontology International*.

[44] Wakefield M. A., Loken B., Hornik R. C. (2010). Use of mass media campaigns to change health behaviour. *The Lancet*.

[45] Wangberg S., Andreassen H., Kummervold P., Wynn R., Sørensen T. (2009). Use of the internet for health purposes: trends in Norway 20002010. *Scandinavian Journal of Caring Sciences*.

[46] Keyes K. M., Utz R. L., Robinson W., Li G. (2010). What is a cohort effect? Comparison of three statistical methods for modeling cohort effects in obesity prevalence in the United States, 19712006. *Social Science & Medicine*.

[47] Heo J., Jeon S.-Y., Oh C.-M., Hwang J., Oh J., Cho Y. (2017). The unrealized potential: cohort effects and age-period-cohort analysis. *Epidemiology and Health*.

